# Potential Range Shifts of Two Sympatric *Fagus* Species

**DOI:** 10.1002/ece3.72979

**Published:** 2026-01-30

**Authors:** Yifeng Chen, Chang Guo, Linlin Cao, Zhixiang Zhang, Wenpan Dong

**Affiliations:** ^1^ School of Ecology and Nature Conservation Beijing Forestry University Beijing China

**Keywords:** climate change, *Fagus*, habitat suitability, MaxEnt model, species distribution modeling

## Abstract

*Fagus longipetiolata* Seemen and *Fagus lucida* Rehder & E.H. Wilson are the dominant species in subtropical deciduous broad‐leaved forests of China, playing crucial ecological and economic roles. As ecologically and economically important trees, it is critical to understand their responses to climate change. This study employed MaxEnt modeling to discover the range shifts from their historical distributions to future projections. The distribution of suitable habitats of *F. longipetiolata* is more affected by precipitation, and that of *F. lucida* is more sensitive to temperature. High‐suitability habitats for both species were concentrated predominantly in the Yangtze River Basin. While both species showed substantial distribution centroid shifts since the Last Glacial Maximum (LGM), *F. lucida* exhibited greater habitat fragmentation and more pronounced reductions in high‐suitability areas under projected climate change compared to *F. longipetiolata*. Our results suggest that despite close phylogenetic relationships, these sister species face divergent climate change threats, with *F. lucida* being more vulnerable. These findings not only advance the conservation strategies for *F. lucida* but also provide critical insights for mitigating the impacts of intensifying global warming on subtropical forest ecosystems.

## Graphical abstract

Using MaxEnt modeling, we found that *Fagus longipetiolata*'s habitat suitability is primarily precipitation‐driven while *F. lucida* 's depends more on temperature, with both species concentrated in the Yangtze River Basin; despite their shared ancestry, *F. lucida* exhibits greater vulnerability to future warming due to accelerated habitat fragmentation and suitability loss, underscoring urgent conservation priorities for subtropical forest resilience.

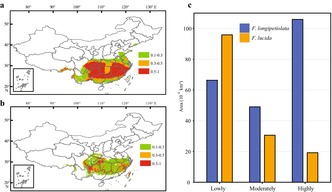

## 1 Introduction

*Fagus* (Fagaceae) is a relatively primitive genus, which is widely distributed in temperate and subtropical regions of the Northern Hemisphere (Denk 2003; Fang and Lechowicz 2006; Hukusima et al. 2013; Shen 1992; Zhang et al. 2013). It is one of the founding species of temperate deciduous broad‐leaved forests, and it is a key component of climax vegetation. Most species are tall trees with durable wood, serving as valuable raw materials. *F. longipetiolata* and *F. lucida* are widely distributed in China, and the current distribution areas of the two species overlap widely in subtropical China (Liu et al. 2003; Shen et al. 2015; Zhang et al. 2013). Phylogenetic relationship evidence of 28 single/low copy nuclear gene loci indicated both species are sister groups and had recent divergence (~5.8 Ma) (Zhang et al. 2013).

During the Last Glacial Maximum (LGM), deciduous broad‐leaved forests dominated by *Fagus* and *Alnus* were widely distributed across subtropical China (Yue et al. 2012). By the early Holocene, these deciduous forests transitioned to evergreen broad‐leaved ecosystems (Lee and Liew 2010; Wan et al. 2021). In recent decades, climate change has increasingly impacted subtropical forests in China, with drought emerging as a primary stressor (Du et al. 2019; Li et al. 2021; Su et al. 2021; Yan et al. 2023; Yin et al. 2024). Notably, *Fagus* species—key dominant taxa in these forest communities—exhibit high sensitivity to drought (Cao et al. 1995; Scharnweber et al. 2011; Yagihashi et al. 2007). While *Fagus lucida* currently lacks formal conservation status on the IUCN Red List of Threatened Species, its congener *F. longipetiolata* is classified as Vulnerable (https://www.iucnredlist.org; accessed March 30, 2025). Recent studies highlight divergent climate responses among sister species with pronounced habitat differentiation (Hu et al. 2025). However, research remains scarce on the climate response of sister species with overlapping habitat distribution. This gap underscores the critical need to investigate the contrasting climatic sensitivities of *F. longipetiolata* and *F. lucida* . Such comparative studies would not only advance our understanding of sister‐species adaptation under climate change but also inform conservation strategies to mitigate climate‐driven threats to China's subtropical forests.

Species distribution models (SDMs) have emerged as a powerful tool in ecological and biogeographical research (Benavides Rios et al. 2024; Safdar et al. 2025), enabling researchers to predict the spatial and temporal patterns of species occurrence based on environmental variables. These models are particularly valuable for understanding the relationships between species and their habitats, as well as for projecting how species distributions may change under future climate change scenarios. Among the various SDM approaches, the Maximum Entropy modeling (MaxEnt) method has gained widespread popularity due to its ability to produce accurate and reliable predictions even with limited occurrence data (Fan and Luo 2025; Khalaf et al. 2024; Ray et al. 2025; Zhao et al. 2024). The MaxEnt model is based on the principle of maximum entropy (Phillips et al. 2006), which aims to maximize the entropy in the probability distribution of species occurrence while incorporating constraints derived from environmental variables. This method has been widely adopted in ecological studies because of its flexibility, robustness, and ability to handle complex ecological relationships. Compared to other SDM approaches, MaxEnt has demonstrated superior performance in modeling species distributions under both current and future climate scenarios (Elith et al. 2015).

In this study, we employ the MaxEnt model to simulate the suitable habitats of two widely distributed *Fagus* sister species, *F. longipetiolata* and *F. lucida* , across different historical periods and future climatic projections. By integrating species occurrence records with environmental variables, this approach provides a robust framework for understanding the ecological drivers of species distributions and for predicting how these species may respond to ongoing climate change. This study is designed to address four pivotal questions: (1) Which bioclimatic variables predominantly constrain the distribution of suitable habitats for *F. longipetiolata* and *F. lucida* , and what are the similarities and differences in their climate responses? (2) What are the current geographic distributions of suitable habitats for *F. longipetiolata* and *F. lucida* ? (3) How have these suitable habitat patterns shifted during the Quaternary period, and how might they evolve under future climate change scenarios? This study will elucidate the cross‐temporal climate adaptation mechanisms of *Fagus* by integrating Quaternary climate fluctuations with predictive modeling under future climate scenarios and develop evidence‐based conservation frameworks informing conservation prioritization and sustainable management practices for this keystone genus within subtropical forest ecosystems of China.

## 2 Materials and Methods

### 2.1 Data Collection and Processing of Distribution Records

Distribution data for *F. longipetiolata* and *F. lucida* were obtained from the Chinese Virtual Herbarium (CVH, http://www.cvh.ac.cn/, last accessed on August 24, 2024) which compiles digitized specimen records from herbaria across China, spanning the period 1902–2023, and from field surveys. All specimens were verified as taxonomically identified records. We excluded records that lacked specimen photographs, had no precise locality descriptions within China, or were apparent misidentifications, in addition to removing duplicates. For samples lacking coordinate information, GPS coordinates were determined by georeferencing the textual locality descriptions (primarily place names and mountain names) using a GPS positioning system and online gazetteers. It should be noted that this georeferencing process, based on textual descriptions, may introduce some positional errors and is a recognized limitation of using historical specimen data. In order to minimize spatial sampling bias caused by clustered occurrence records, SDM Toolbox v2.5 in ArcGIS v10.8 was used to filter the data, and only one distribution record was kept for each 10 km × 10 km grid cell. Ultimately, 94 valid records for *F. longipetiolata* and 77 for *F. lucida* were obtained (Figure S1, Table S1). These records represent species presence points; detailed ecological metadata (e.g., population size, tree growth data) are typically not available in such specimen databases.

### 2.2 Bioclimatic Variables

Environmental data for MaxEnt modeling were derived from the WorldClim database (http://www.worldclim.org/; accessed July 5, 2024) at a spatial resolution of 2.5 min. Current climate data (1970–2000) were obtained from the Coupled Model Intercomparison Project Phase 6 (CMIP6) for 19 bioclimatic variables (bio1–bio19; for full definitions, see Table S2). Future climate data for the 2070s (2060–2080) under the SSP585 emission scenario were also used. In addition, historical climate data for the Last Glacial Maximum (~21 ka BP) and Mid‐Holocene (~6 ka BP) were obtained from the Community Climate System Model version 4 (CCSM4). ArcGIS was employed to extract the values of the 19 bioclimatic variables at each occurrence record. Correlation analysis was conducted using SPSS, and environmental factors with higher contribution rates were selected among variables with pairwise correlation coefficients |*r*| > 0.8. Finally, 10 bioclimatic factors of *F. longipetiolata* and 8 bioclimatic factors of *F. lucida* were selected (Tables 1 and 2).

**TABLE 1:** Ten bioclimatic variables of *F. longipetiolata* contributions and permutation importance in the MaxEnt model.

Environmental variable	Percent contribution (%)	Permutation importance (%)
bio16 (Precipitation of Wettest Quarter)	58.2	40.1
bio4 (Temperature Seasonality)	15.0	7.5
bio11 (Mean Temperature of Coldest Quarter)	14.1	8.6
bio14 (Precipitation of Driest Month)	4.2	1.3
bio9 (Mean Temperature of Driest Quarter)	3.3	14.3
bio6 (Min Temperature of Coldest Month)	2.8	11.4
bio1 (Annual Mean Temperature)	1.2	14.1
bio10 (Mean Temperature of Warmest Quarter)	0.5	0.3
bio17 (Precipitation of Driest Quarter)	0.4	0.7
bio12 (Annual Precipitation)	0.2	1.7

**TABLE 2:** Eight bioclimatic variables of *F. lucida* contributions and permutation importance in the MaxEnt model.

Environmental variable	Percent contribution (%)	Permutation importance (%)
bio16 (Precipitation of Wettest Quarter)	54.1	15.2
bio2 (Mean Diurnal Range)	14.2	18.4
bio10 (Mean Temperature of Warmest Quarter)	8.1	3.4
bio11 (Mean Temperature of Coldest Quarter)	7.9	36.2
bio18 (Precipitation of Warmest Quarter)	5.9	12.8
bio15 (Precipitation Seasonality)	4.0	1.7
bio4 (Temperature Seasonality)	3.3	11.9
bio8 (Mean Temperature of Wettest Quarter)	2.7	0.5

### 2.3 MaxEnt Model Simulations and Evaluation

The MaxEnt v3.4.1 was used to simulate the historical, current, and future distributions of *F. longipetiolata* and *F. lucida* (Phillips et al. 2004). The model settings included the activation of a random seed, a test dataset comprising 25% of the records (with the remaining 75% used for training), a maximum of 10,000 background points, 10 replicate runs using the Bootstrap method, and a maximum iteration count of 5000. Other parameters were kept at their default values. The model output was generated in logistic format (Phillips and Dudík 2008), and response curves along with jackknife tests were computed to assess variable importance. AUC value close to 1 indicates a close correlation between bioclimatic variables and the predicted geographical distribution of species. In general, when the AUC value is greater than 0.9, the simulation results are more accurate, while when the AUC value is lower than 0.6, the simulation results are not reliable (Swets 1988).

### 2.4 Habitat Suitability Classification

The habitat suitability values ranged from 0 to 1, with higher values indicating a greater likelihood of species occurrence. Based on the Jenks Natural Breaks classification (Jenks 1967), the study area was divided into four categories using ArcGIS v10.8: unsuitable habitat (0–0.1), lowly suitable habitat (0.1–0.3), moderately suitable habitat (0.3–0.5), and highly suitable habitat (0.5–1). The Zonal Geometry as Table in ArcGIS v10.8 was used to calculate the suitable habitat area. Finally, the maps and areas of suitable habitats of *F. longipetiolata* and *F. lucida* in each time period and under each climate scenario were obtained, enabling subsequent comparative analyses.

### 2.5 Migration and Change of Suitable Habitat

Based on the distribution maps generated for the past, present, and future climate scenarios (SSP126 and SSP585), the Mean Center tool in ArcGIS v10.8 was applied to calculate the centroid of highly suitable habitats for each time period. These centroids represent the overall spatial locations of highly suitable habitat and were used to infer shift directions and distances over time. Additionally, the expansion and contraction of suitable areas from the current period (1970–2000) to the 2070s (2061–2080) were evaluated under both climate scenarios.

## 3 Results

### 3.1 Bioclimatic Variables

The MaxEnt model produced ROC curves based on 94 records for *F. longipetiolata* (10 bioclimatic variables) and 77 records for *F. lucida* (8 bioclimatic variables). The average training AUC values over 10 replicates were 0.993 for *F. longipetiolata* and 0.962 for *F. lucida* (Figure S2a), indicating high model accuracy (Fielding and Bell 1997; Pearce and Ferrier 2000).

For *F. longipetiolata*, jackknife analysis revealed that when used individually, the top three variables in terms of regularized training gain were Mean Temperature of Driest Quarter (bio9), Mean Temperature of Coldest Quarter (bio11), and Annual Mean Temperature (bio1) (Figure S2b). These variables also yielded the highest gains in test AUC. Omission of Precipitation of Wettest Quarter (bio16), Temperature Seasonality (bio4), and bio9 resulted in the largest declines in training gain, underscoring their unique explanatory power. Similarly, Annual Precipitation (bio12), bio16, and bio9 showed the greatest reductions in test when excluded.

In *F. lucida* , the results showed the highest regularized training gains were bio16, Precipitation of Warmest Quarter (bio18), and Mean Diurnal Range (bio2) (Figure S2b). In the AUC, bio16, bio18, and bio2 were also the highest values. Bio11, bio18, and bio2 were the top three values in the test. In the regularized training gain, it indicated that bio11, bio16, and bio2 were most critical, with the largest drop in gain occurring upon the removal of these variables, suggesting they provide unique information not presented by other bioclimatic variables. In the test, Mean Temperature of Warmest Quarter (bio10), bio2, and bio4 had the largest decrease, while in the AUC it was bio10, bio11, and bio2, which highlights their substantial contribution to model robustness and predictive stability.

### 3.2 Current Suitable Habitat

**FIGURE 1:**
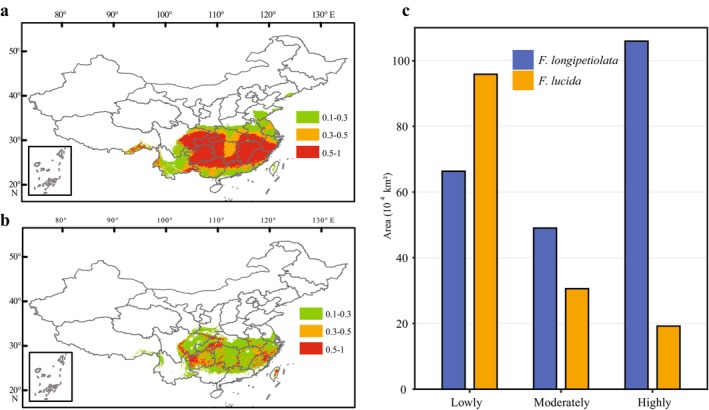
Current suitable habitats of *F. longipetiolata* and *F. lucida* . (a) The current suitable habitats of *F. longipetiolata* modeled with ten variables. (b) The current suitable habitats of *F. lucida* modeled with eight variables. (c) Areas of lowly suitable habitat, moderately suitable habitat, and highly suitable habitat of both species.

The simulation results indicated that suitable habitats for both species were primarily located in southern China. The moderate‐ and high‐suitability habitats of *F. longipetiolata* were relatively concentrated, primarily distributed in: eastern Sichuan; Chongqing; southwestern and southeastern Hubei; southern Anhui; southern Zhejiang; Fujian; Jiangxi; southern and western Hunan; Guizhou; northern Guangdong; and northern Guangxi (Figure 1a). In contrast, the moderate‐ and high‐suitability habitats of *F. lucida* were scattered across south‐central Sichuan; southeastern Chongqing; southwestern Hubei; southern Anhui; southern Zhejiang; northeastern Fujian; northeastern Jiangxi; southwestern Hunan; western Guizhou; and central and eastern Taiwan (Figure 1b).

The total area of suitable habitat for *F. longipetiolata* was 221.26 × 10^4^ km^2^, comprising 66.32 × 10^4^ km^2^ of low‐suitability, 49.04 × 10^4^ km^2^ of moderate‐suitability, and 105.90 × 10^4^ km^2^ of high‐suitability areas. *F. lucida* occupied a total suitable area of 145.66 × 10^4^ km^2^, with 95.85 × 10^4^ km^2^ classified as low‐suitability, 30.60 × 10^4^ km^2^ as moderate‐suitability, and 19.21 × 10^4^ km^2^ as high‐suitability (Figure 1c).

### 3.3 Past Suitable Habitat

**FIGURE 2:**
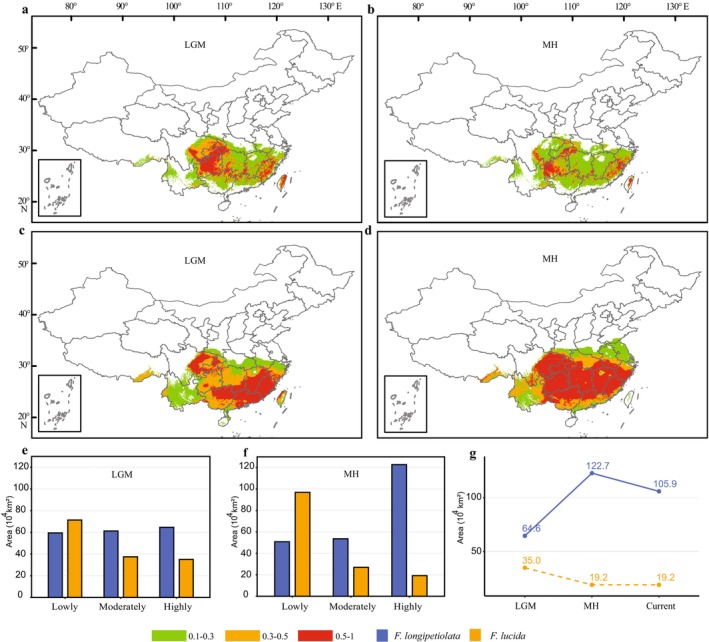
Historical changes in suitable habitat for *F. longipetiolata* and *F. lucida* . (a, b) The suitable habitat of *F. lucida* during the Last Glacial Maximum (a) and mid‐Holocene (b). (c, d) The suitable habitat of *F. longipetiolata* during the Last Glacial Maximum (c) and mid‐Holocene (d). (e, f) The area of lowly suitable habitat, moderately suitable habitat, and highly suitable habitat during Last Glacial Maximum (e) and mid‐Holocene (f). (g) The area of highly suitable habitat during different periods of both species.

During the Last Glacial Maximum (LGM), the total suitable area for *F. longipetiolata* was 185.36 × 10^4^ km^2^, with low‐, moderate‐, and high‐suitability areas of 59.49 × 10^4^ km^2^, 61.26 × 10^4^ km^2^, and 64.61 × 10^4^ km^2^, respectively. In the Mid‐Holocene (MH), the total area increased to 227.19 × 10^4^ km^2^, distributed as 50.84 × 10^4^ km^2^ (low), 53.61 × 10^4^ km^2^ (moderate), and 122.74 × 10^4^ km^2^ (high). For *F. lucida* , the LGM suitable area was 143.90 × 10^4^ km^2^ (71.37 × 10^4^ km^2^ low, 37.48 × 10^4^ km^2^ moderate, 35.05 × 10^4^ km^2^ high), and during the MH, it was 143.07 × 10^4^ km^2^ (96.89 × 10^4^ km^2^ low, 26.97 × 10^4^ km^2^ moderate, 19.21 × 10^4^ km^2^ high) (Figure 2e–g).

The distribution pattern of historical suitable habitats of *F. longipetiolata* changed significantly compared with that of current suitable habitats (Figure 1a), while the distribution pattern of *F. lucida* remained nearly stable. During the Last Glacial Maximum, the high‐suitability habitats of *F. longipetiolata* were mainly concentrated in southern Hunan, southern Jiangxi, Fujian, northern Guangxi, northern Guangdong, eastern Sichuan, and Chongqing. From the Last Glacial Maximum to the Mid‐Holocene, the distribution area of high‐suitability habitat of *F. longipetiolata* was significantly expanded, and was basically consistent with the current distribution pattern of suitable habitat except in central and northern Hunan. The result reveals contrasting spatiotemporal patterns in high‐suitability habitats between the two species (Figure 2a–d). *F. longipetiolata* exhibited an initial range expansion followed by contraction from the Last Glacial Maximum (LGM) to the present, whereas *F. lucida* demonstrated continuous habitat reduction until the mid‐Holocene, after which its suitable area stabilized through to modern times.

### 3.4 Future Suitable Habitat

**FIGURE 3:**
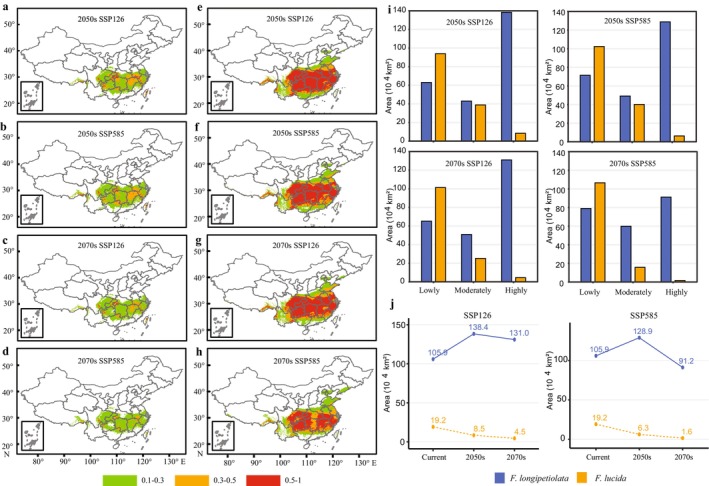
Suitable habitats of *F. lucida* and *F. longipetiolata* under the future climate. (a–d) The suitable habitat of *F. lucida* during the 2050s and 2070s under SSP585 and SSP126. (e–h) The suitable habitat of *F. longipetiolata* during the 2050s and 2070s under SSP585 and SSP126. (i) The area of lowly suitable habitat, moderately suitable habitat, and highly suitable habitat of both species during the 2050s and 2070s under SSP585 and SSP126. (j) The area of highly suitable habitat changes of both species in different periods under SSP585 and SSP126.

Compared with the current suitable habitat (Figure 1), the pattern of suitable habitat of *F. longipetiolata* under the SSP126 and SSP585 scenarios had a great change (Figure 3e–h), showing a certain poleward expansion. Under SSP126, the high‐suitability areas were projected to increase from 105.90 × 10^4^ km^2^ (current) to 138.40 × 10^4^ km^2^ in the 2050s before declining to 131.00 × 10^4^ km^2^ in the 2070s. Under SSP585, the corresponding high‐suitability areas were expected to be 105.90 × 10^4^ km^2^ (current), 128.90 × 10^4^ km^2^ (2050s), and 91.20 × 10^4^ km^2^ (2070s) (Figure 3i,j).

In contrast, *F. lucida* was predicted to experience a continuous reduction in high‐suitability habitats (Figure 3a–d): from 19.21 × 10^4^ km^2^ (current) to 8.5 × 10^4^ km^2^ (2050s) and 4.5 × 10^4^ km^2^ (2070s) under SSP126, and to 6.3 × 10^4^ km^2^ (2050s) and 1.6 × 10^4^ km^2^ (2070s) under SSP585 (Figure 3i,j). In the two future scenarios, the area of moderately and highly suitable habitat of *F. longipetiolata* would be larger than that of *F. lucida* , while the area of lowly suitable habitat will be relatively small. The change of the area of highly suitable habitat of *F. longipetiolata* from the present to the future would first increase and then decrease under both future climate scenarios. In the future scenario of SSP126, the area of highly suitable habitat for both species would be larger than that of SSP585. Figure 3 detailed these trends, highlighting a marked change in suitable habitat for both species, with *F. lucida* being more adversely affected.

### 3.5 Changes in Suitable Habitats

**FIGURE 4:**
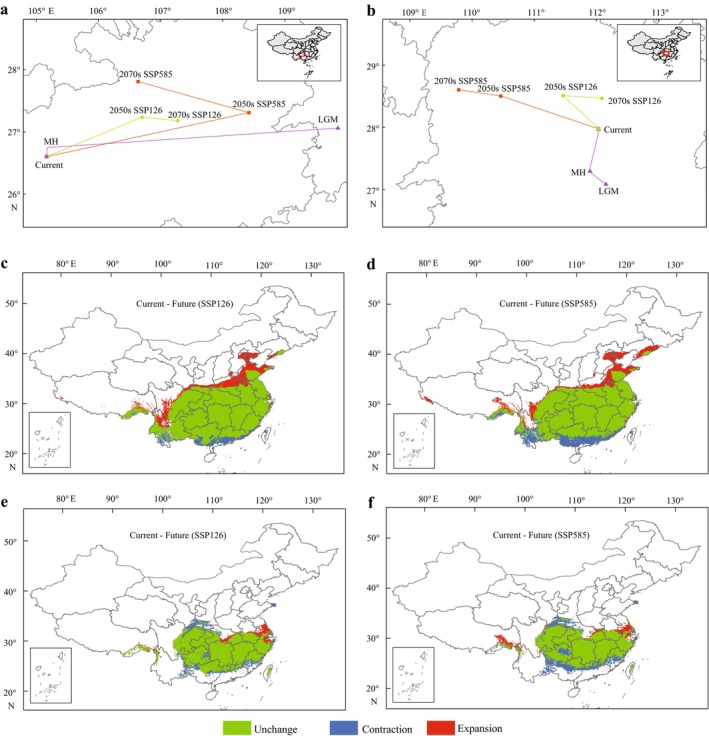
The Centroid migration and changes in suitable habitat. (a, b) Centroid migration of *F. longipetiolata* (a) and *F. lucida* (b). (c, d) Comparison of habitat expansion and contraction of *F. longipetiolata* in the Current period and 2070s under SSP126 and SSP585. (e, f) Comparison of habitat expansion and contraction of *F. lucida* in the Current period and 2070s under SSP126 and SSP585.

The centroid of high‐suitability habitat for *F. longipetiolata* shifted from Hunan (during the LGM) to Guizhou (current), followed by northward shifts under SSP126 and SSP585 scenarios (Figure 4a). Notably, the centroid migration distance of *F. longipetiolata* from LGM to the MH was the longest. *F. lucida* also exhibited a persistent northward migration, with its centroid moving within Hunan province from the LGM to the 2070s (Figure 4b). Furthermore, areas in southern Guangxi, Guangdong, and Yunnan currently suitable for *F. longipetiolata* might become unsuitable in future scenarios (Figure 4c,d), while *F. lucida* could lose suitable habitats in southern Gansu and Shanxi (Figure 4e,f). Overall, both species were predicted to migrate northward, with *F. lucida* showing more pronounced habitat contraction.

## 4 Discussion

### 4.1 Bioclimatic Variables Affecting the Suitable Habitat of *F. longipetiolata* and *F. lucida*

*Fagus longipetiolata* and *F. lucida* are both species of *Fagus* widely distributed in China. Most of these plants are sensitive to drought. The differentiation of the two ecological niches is not obvious (Cai et al. 2021; Li et al. 2024), which is consistent with the results of this study. The MaxEnt simulation results based on current climate data showed (Tables 1 and 2) that the bioclimatic variables with the greatest contribution rates to predict the distribution of suitable habitats of *F. longipetiolata* and *F. lucida* were the same, bio16, which was Precipitation of Wettest Quarter related to precipitation during the breeding or growth season. This result showed that the primary limitation on suitable habitat distribution was precipitation.

However, it is worth noting that bioclimatic variable bio16 was the most important for *F. longipetiolata* both in terms of contribution rates and permutation importance values, which were 58.2% and 40.1% respectively. For *F. lucida* , although the contribution rates of bioclimatic variable bio16 were the largest, which was 54.1%, the permutation importance value was only 15.2%. For *F. lucida* , the bioclimatic variable with the largest permutation importance values was bio11 (Mean Temperature of Coldest Quarter), which was 36.2%, indicating that the importance of bio11 in predicting suitable habitat for *F. lucida* had become significantly greater. The high PI value implied its irreplaceable role in limiting the survival range of species, especially in the case of cold stress. Previous studies have shown that winter low temperature was likely to be an important threshold climate variable for *Fagus*, as the isotherm derived using the 1% quantile of the bioclimatic variable of winter temperature almost coincides with the northern boundary of *Fagus* (Wan et al. 2023). This well explains why the bioclimatic variable with the largest PI value was bio11, whose threshold action leads to high PI values. The effect of low temperature on *F. lucida* is likely to be the same as that of other plants by influencing their growth (Bai et al. 2019; Lyu et al. 2020; Panthi et al. 2018) or seedling survival (Carroll et al. 2021) to influence the distribution of suitable habitats. In addition, *F. longipetiolata* was more affected by temperature when variables were used alone according to the jackknife results. For example, the top three highest values in regularized training gain, test and AUC were bio9, bio11 and bio1. Moreover, *F. lucida* was more affected by precipitation in jackknife results, such as bio16 and bio18.

### 4.2 Changes of Suitable Habitat Distribution of *F. longipetiolata* and *F. lucida*

The highly suitable habitat regions of *F. longipetiolata* changed significantly, shifting from a north–south discontinuous distribution during the Last Glacial Maximum (Figure 2c), to an expansion range in the mid‐Holocene (Figure 2d), and finally forming an east–west discontinuous pattern in the current period (Figure 1a). The highly suitable habitat areas of *F. lucida* had been decreasing (Figures 2g and 3j), and the distribution pattern of *F. lucida* had been fragmented (Figures 1b, 2a,b, and 3a–d) since the Last Glacial Maximum period. Pollen records of the genus indicated that the distribution range in the late Quaternary was primarily confined to subtropical areas. Only a small amount of pollen appeared along the northern part of the coastline in the mid‐Holocene, but it disappeared by the current period (Wan et al. 2023). During the Last Glacial Maximum period, the highly suitable habitat in the southeast of *F. longipetiolata* was distributed along most of the coastline of southern Guangdong and all of the coastline of Fujian (Figure 2c), while after the mid‐Holocene period, the highly suitable habitat in the southeast of *F. longipetiolata* moved northward (Figure 2d). Only a small part of the coastline in northeast Fujian and the coastline in southeast Zhejiang remained highly suitable habitats and had remained so until the current period (Figure 1a). However, the reason why the pollen records appeared in the southeastern coastline had disappeared in the current period might be due to human activities rather than climate change (Cao et al. 1995). The predicted distribution patterns aligned with previous pollen studies, indicating model reliability. Previous studies had shown that the haplotype lineages of the current *F. lucida* population were distributed in a mosaic pattern, which was probably due to the long‐term isolation of mountain regions during the Quaternary period (Zhang et al. 2013), and our study also confirmed that the distribution pattern of highly suitable habitat in the central and eastern part of *F. lucida* had been in a fragmented state since the Last Glacial Maximum stage. The northward migration of *Fagus* inferred from pollen records (Wan et al. 2023) aligned with our model projections, further validating the reliability of the results.

Under SSP126 and SSP585 scenarios, the future suitable habitat distribution patterns of *F. longipetiolata* and *F. lucida* were predicted in the 2050s and 2070s. SSP126 and SSP585 represent the lowest and highest greenhouse gas emission scenarios, respectively. From now to the 2050s and then to the 2070s, the highly suitable areas of *F. longipetiolata* under both climate scenarios showed a trend of first increasing and then decreasing (Figure 3j). This suggested that future climate change, due to greenhouse gases such as carbon dioxide emitted by human activities, might initially enhance photosynthesis in *F. longipetiolata*, favoring their growth and development. However, if temperatures continued to rise, excessive heat stress could negate these benefits, as seen in the east–west fragmentation of its high‐suitability habitat under SSP585 by the 2070s (Figure 3h). In the future, the highly suitable habitat of *F. lucida* was in a shrinking state under both climate scenarios, and not only from the current period to the future, but also from the past Last Glacial Maximum period. Although the moderately suitable habitat of *F. lucida* obviously expanded in the northern part of Jiangxi from the current period to the 2050s, it began to shrink gradually from the 2050s to 2070s. Under the SSP585 scenario, all highly and moderately suitable habitats of *F. lucida* had almost disappeared by the 2070s (Figure 3a–d,i,j).

In addition, under both climate scenarios, the MaxEnt model showed that the overall migration trend of *F. longipetiolata's* centroid in central and southern China's Guizhou was northeastward (Figure 4a). However, under the SSP585 scenario, the centroid shifted northwestward from the 2050s to the 2070s. Predictions revealed that the centroid migration distance of *F. longipetiolata* under SSP585 was significantly longer than under SSP126. For *F. lucida* , the general migration trend under both future scenarios was northwestward, but under SSP126, the centroid shifted southeastward from the 2050s to the 2070s. The migration distance under SSP585 was also longer than under SSP126.

The centroid migration of *F. longipetiolata* revealed a long‐distance shift from western Hunan (LGM) to central and western Guizhou (MH), the longest migration distance compared to other centroid shifts of this species (Figure 4a). This might be due to its highly suitable habitat in the southern part of the Last Glacial Maximum stage and its gradual northward expansion during the mid‐Holocene stage, merging with habitats in Sichuan and Chongqing (Figure 2c,d). The distribution center of *F. longipetiolata* has been in Guizhou since it moved from Hunan to Guizhou in the Last Glacial Maximum period, while the distribution center of *F. lucida* has been in Hunan.

Comparisons of habitat expansion/contraction between the current period and the 2070s (Figure 4c–f) suggested that eastern Tibet would become a new suitable habitat for both species. Under the scenario of SSP585, the area of contraction (blue regions) was substantially larger than under SSP126. *F. longipetiolata* exhibited pronounced northward shifts in both upper and lower boundaries (Figure 4c,d). In contrast, *F. lucida* 's lower boundary showed minimal northward movement, while its upper boundary partially shifted from Zhejiang to Jiangsu. Populations in Shanxi (upper boundary) experienced contraction, potentially due to limitations imposed by the East Asian monsoon on the northward expansion of *F. lucida* (Wan et al. 2023).

Both species consistently migrated northward across all climate scenarios, whether considering distribution centers or range boundaries. This was consistent with the results of many other studies (Lima et al. 2024; Luo et al. 2024; Shi et al. 2024; Yin et al. 2025; Zhang et al. 2024, 2022). Under global warming, many plants generally showed a trend of migration to higher latitudes.

### 4.3 Methodological Considerations and Future Directions

In this study, we utilized species occurrence records from the Chinese Virtual Herbarium (CVH) to model large‐scale, climate‐driven potential distribution shifts. Such records provide the broad geographic coverage necessary for assessing climatic niches, though they typically lack fine‐scale ecological metadata (e.g., stand details or growth measurements). Correspondingly, our modeling approach focuses on climatic suitability by isolating the influence of climatic drivers, while acknowledging that other factors such as species interactions and anthropogenic factors also shape distributions at local scales. Finally, as a probabilistic and nonlinear modeling framework, our approach is designed to identify regions of climatic suitability potential rather than to provide deterministic predictions of precise species distributions validated with growth data. While these considerations mean that our maps represent the fundamental climatic niche, they establish a critical baseline. Future studies that integrate plot‐based growth data, process‐based models, and additional biotic filters will be essential for refining these projections toward the realized niche.

## 5 Conclusion

The MaxEnt modeling approach successfully simulated the distribution patterns of *Fagus longipetiolata* and *Fagus lucida* across historical, current, and future climatic scenarios. The results indicated that high‐suitability habitats for both species were predominantly concentrated in the Yangtze River Basin, with Precipitation of Wettest Quarter (bio16) emerging as the key bioclimatic variable. Notably, although both species shared the same key bioclimatic variable, *F. lucida* exhibited a greater sensitivity to Mean Temperature of Coldest Quarter (bio11) and experienced more pronounced habitat fragmentation and contraction over time. These differential responses suggest that *F. lucida* is likely to face higher risks under future climate change scenarios compared to *F. longipetiolata*. In light of these findings, targeted conservation and sustainable management strategies should be prioritized for *F. lucida* . Future research should further investigate the mechanistic basis of these species‐specific responses to refine conservation efforts for the significant genus *Fagus*, and provide critical insights for mitigating the impacts of intensifying global warming on subtropical forest ecosystems.

## Author Contributions

**Yifeng Chen:** formal analysis (equal), software (equal), writing – original draft (equal), writing – review and editing (equal). **Chang Guo:** formal analysis (equal), software (equal). **Linlin Cao:** data curation (equal), software (equal). **Zhixiang Zhang:** conceptualization (equal), project administration (equal), supervision (equal), writing – original draft (equal), writing – review and editing (equal). **Wenpan Dong:** conceptualization (equal), funding acquisition (equal), project administration (equal), supervision (equal), writing – original draft (equal), writing – review and editing (equal).

## Conflicts of Interest

The authors declare no conflicts of interest.

## Supporting information


**Figure S1:** 94 occurrence records of *F. longipetiolata* and 77 of *F. lucida* .

**Figure S2:** MaxEnt Simulations and Model Accuracy Evaluation. (a) Receiver operating characteristic (ROC) curves of *Fagus longipetiolata* and *Fagus lucida*. (b) Jackknife test of variable importance of *Fagus longipetiolata* and *Fagus lucida*.

**Table S1:** 94 occurrence records of *F. longipetiolata* and 77 of *F. lucida* .

**Table S2:** Descriptions of the 19 bioclimatic variables.

## Data Availability

The species occurrence records used in this study were sourced from the publicly accessible Chinese Virtual Herbarium (CVH, http://www.cvh.ac.cn/). The bioclimatic variables were obtained from the WorldClim database (http://www.worldclim.org/). The cleaned occurrence dataset (latitude and longitude) used for the final MaxEnt modeling is available in Supporting Information (Table S1).
