# Supplementary figures and images for: Potential Range Shifts of Two Sympatric Fagus Species
Source: Ecol Evol. 2026 Jan 30;16(2):e72979. doi: 10.1002/ece3.72979 (PMC12856369; doi:10.1002/ece3.72979)

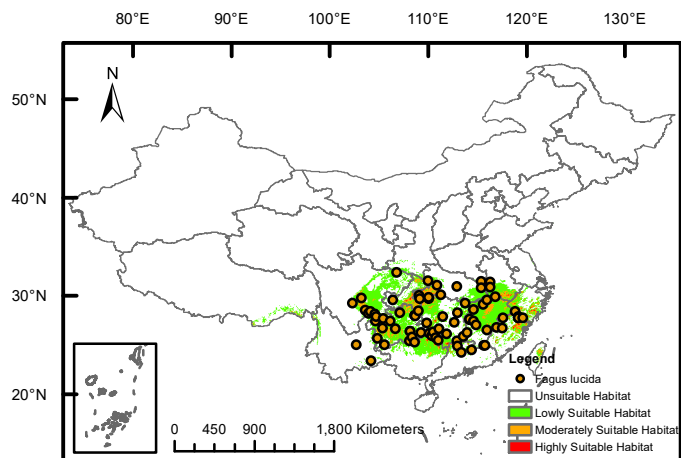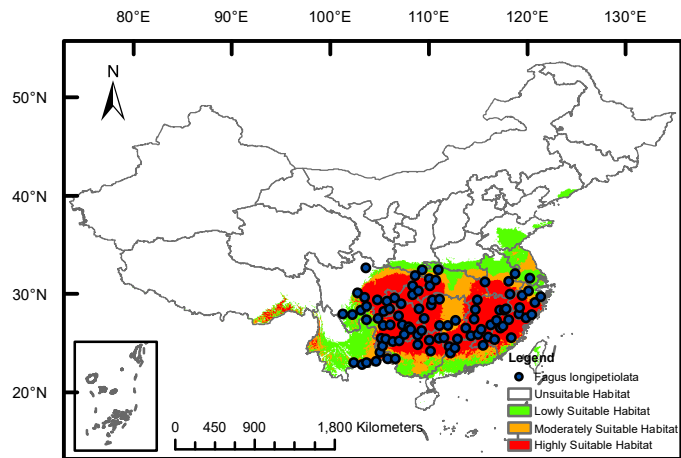

Supplement: Supplementary file 1 — Figure S1: 94 occurrence records of F. longipetiolata and 77 of F. lucida . [file ECE3-16-e72979-s001.pdf]

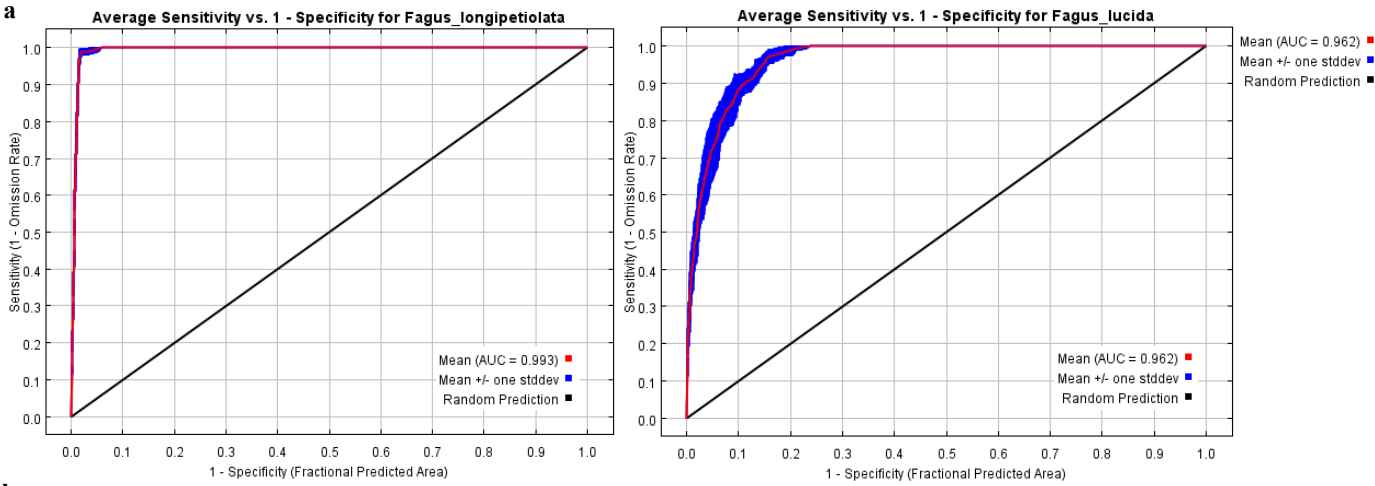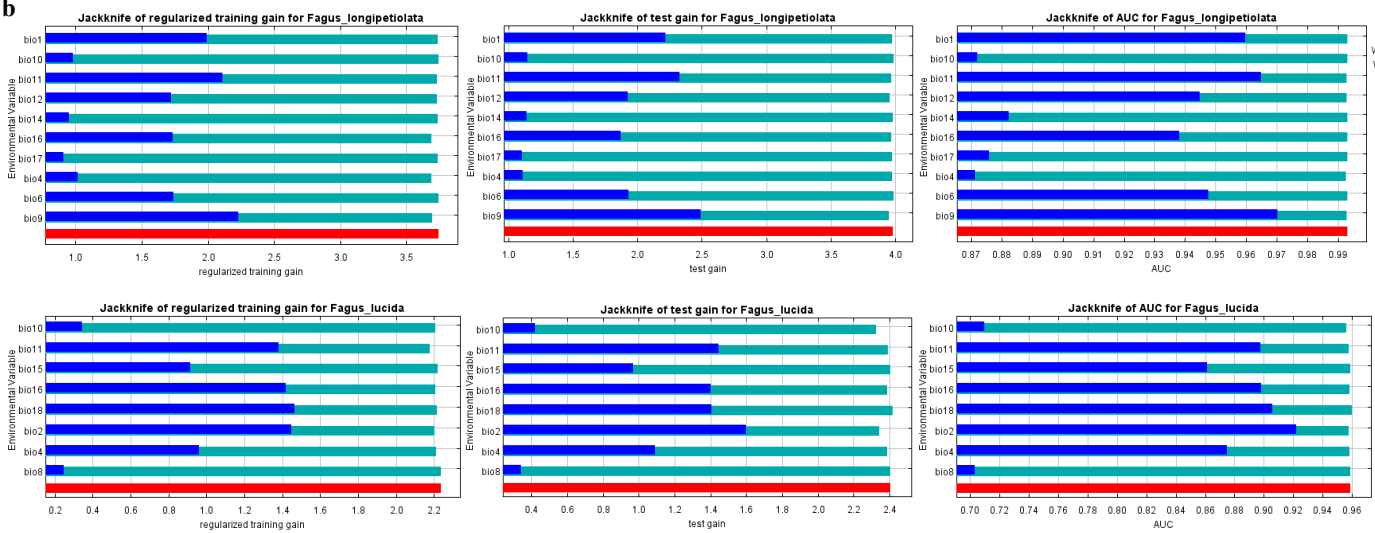

Supplement: Supplementary file 2 — Figure S2: MaxEnt Simulations and Model Accuracy Evaluation. (a) Receiver operating characteristic (ROC) curves of Fagus longipetiolata and Fagus lucida. (b) Jackknife test of variable importance of Fagus longipetiolata and Fagus lucida. [file ECE3-16-e72979-s003.pdf]
