# Supplementary material for: Potential Range Shifts of Two Sympatric Fagus Species
Source: Ecol Evol. 2026 Jan 30;16(2):e72979. doi: 10.1002/ece3.72979 (PMC12856369; doi:10.1002/ece3.72979)
Supplement: Supplementary file 4 — Table S2: Descriptions of the 19 bioclimatic variables. [file ECE3-16-e72979-s004.docx]

Table S2. Descriptions of the 19 bioclimatic variables

Environmental Variable Description

bio1 Annual Mean Temperature

bio2 Mean Diurnal Range

bio3 Isothermality (bio2/bio7) (*100)

bio4 Temperature Seasonality (standard deviation *100)

bio5 Max Temperature of Warmest Month

bio6 Min Temperature of Coldest Month

bio7 Temperature Annual Range (bio5-bio6)

bio8 Mean Temperature of Wettest Quarter

bio9 Mean Temperature of Driest Quarter

bio10 Mean Temperature of Warmest Quarter

bio11 Mean Temperature of Coldest Quarter

bio12 Annual Precipitation

bio13 Precipitation of Wettest Month

bio14 Precipitation of Driest Month

bio15 Precipitation Seasonality (coefficient of variation)

bio16 Precipitation of Wettest Quarter

bio17 Precipitation of Driest Quarter

bio18 Precipitation of Warmest Quarter

bio19 Precipitation of Coldest Quarter
